# A New Integrative and Mobilizable Element Is a Major Contributor to Tetracycline Resistance in *Streptococcus dysgalactiae* subsp. *equisimilis*

**DOI:** 10.3390/antibiotics12030579

**Published:** 2023-03-15

**Authors:** Guillem López de Egea, Aida González-Díaz, Gérard Guédon, Julie Lao, Dàmaris Berbel, Antonio Casabella, José María Marimón, Emilia Cercenado, Lucía Fernández-Delgado, Hélène Chiapello, Thomas Lacroix, María Ángeles Domínguez, Nathalie Leblond-Bourget, Carmen Ardanuy

**Affiliations:** 1 Microbiology Department, Hospital Universitari Bellvitge, IDIBELL-UB, 08907 L’Hospitalet de Llobregat, Spain; 2 Research Network for Respiratory Diseases (CIBERES), Insituto de Salud Carlos III (ISCIII), 28029 Madrid, Spain; 3 DynAMic, Université de Lorraine, Institut national de recherche pour l’agriculture et l’environnement, Faculté des Sciences et Technologies, F-54000 CEDEX Nancy, France; 4 MaIAGE, Université Paris-Saclay, Institut national de recherche pour l’agriculture et l’environnement, Domaine de Vilvert, F-78350 CEDEX Jouy-en-Josas, France; 5 Department of Genetics and Microbiology, Universitat Autònoma de Barcelona, 08193 Barcelona, Spain; 6 Microbiology Department, Hospital Universitari Parc Taulí, 08208 Sabadell, Spain; 7 Biodonostia, Infectious Diseases Area, Respiratory Infection and Antimicrobial Resistance Group, Osakidetza Basque Health Service, Donostialdea Integrated Health Organization, Microbiology Department, 20014 San Sebastián, Spain; 8 Clinical Microbiology and Infectious Disease Department, Hospital General Universitario Gregorio Marañón, 28009 Madrid, Spain; 9 Department of Pathology and Experimental Therapeutics, School of Medicine and Health Sciences, University of Barcelona, 08907 Barcelona, Spain; 10 Research Network for Infectious Diseases (CIBERINFEC), ISCIII, 28029 Madrid, Spain

**Keywords:** *Streptococcus dysgalactiae* subsp. *equisimilis*, *tet*(M), IME, ICE, mobile genetic elements

## Abstract

Tetracycline resistance in streptococci is mainly due to ribosomal protection mediated by the *tet*(M) gene that is usually located in the integrative and conjugative elements (ICEs) of the Tn*916*-family. In this study, we analyzed the genes involved in tetracycline resistance and the associated mobile genetic elements (MGEs) in *Streptococcus dysgalactiae* subsp. *equisimilis* (SDSE) causing invasive disease. SDSE resistant to tetracycline collected from 2012 to 2019 in a single hospital and from 2018 in three other hospitals were analyzed by whole genome sequencing. Out of a total of 84 SDSE isolates, 24 (28.5%) were resistant to tetracycline due to the presence of *tet*(M) (*n* = 22), *tet*(W) (*n* = 1), or *tet*(L) plus *tet*(W) (*n* = 1). The *tet*(M) genes were found in the ICEs of the Tn*916*-family (*n* = 10) and in a new integrative and mobilizable element (IME; *n* = 12). Phylogenetic analysis showed a higher genetic diversity among the strains carrying Tn*916* than those having the new IME, which were closely related, and all belonged to CC15. In conclusion, tetracycline resistance in SDSE is mostly due to the *tet*(M) gene associated with ICEs belonging to the Tn*916*-family and a new IME. This new IME is a major cause of tetracycline resistance in invasive *Streptococcus dysgalactiae* subsp. *equisimilis* in our settings.

## 1. Introduction

*Streptococcus dysgalactiae* subsp. *equisimilis* (SDSE) are beta-hemolytic streptococci that possess cell wall carbohydrate serogroup antigens C and G and sometimes A or L. SDSE are present in the microbiota of the respiratory, gastrointestinal, and female genital tract and the skin of humans [1,2]. It usually causes mild to severe infections in humans as *Streptococcus pyogenes,* ranging from pharyngitis and skin infections to necrotizing fasciitis and streptococcal toxic shock-like syndrome [2,3]. These two species are phylogenetically closely related and share many virulence factors, such as the M-protein, involved in the adhesion and immune system evasion, and used for epidemiological typing [4]. In recent years, two important facts related to SDSE have been described: first, the global emergence of invasive disease due to SDSE [5,6,7,8] and second, the description of the horizontal DNA transfer from SDSE to *S. pyogenes*, such as *pbp2X* gene, leading *S. pyogenes* to have a decreased susceptibility to beta-lactams [9].

Tetracycline resistance can be mediated by three main mechanisms: active efflux pumps, ribosomal protection, and enzymatic inactivation. Within the genus *Streptococcus,* ribosomal protection is the most important mechanism, and the *tet*(M) gene the most frequent. This gene is mostly found in the integrative and conjugative elements (ICEs), mainly of the Tn*916*-family, which can spread through horizontal transmission by conjugation [10]. In fact, having all the requirements for self-transmission is a hallmark of ICEs. On the other hand, integrative and mobilizable elements (IMEs) are elements that encode their own excision and integration, possess only some sequence and genes involved in conjugation, and hijack the conjugation apparatus of conjugative plasmids or ICEs to promote their own transfer [11]. Despite the fact that IMEs’ prevalence is still underestimated, many of them encode resistance to antibiotics and altogether confer resistance to a large variety of antibiotics [11]. Considering resistance to tetracycline, several IMEs from *Streptococcus suis* were found to carry the *tet*(O) gene and one a *tet*(40) gene [12,13,14]. However, no IME carrying *tet*(M) had ever been identified in Firmicutes. During a surveillance study of tetracycline resistance in SDSE we found a new IME carrying the *tet*(M) gene. Hence, the aim of the present study was to describe this new element and to analyze the epidemiology of tetracycline resistance in SDSE.

## 2. Results and Discussion

Out of a total of 84 SDSE isolates causing invasive disease (iSDSE), 24 (28.5%) were resistant to tetracycline. In order to analyze the trends in the iSDSE over the study period, we analyzed data from the Hospital Universitari de Bellvitge (HUB) covering the whole period. Even though the number of isolates collected per year was low, tetracycline resistance rates remained stable among the iSDSE collected from HUB from 37.5% (3/8) in 2012–2015 to 30.2% (16/53) in 2016–2019.

**Figure 1:**
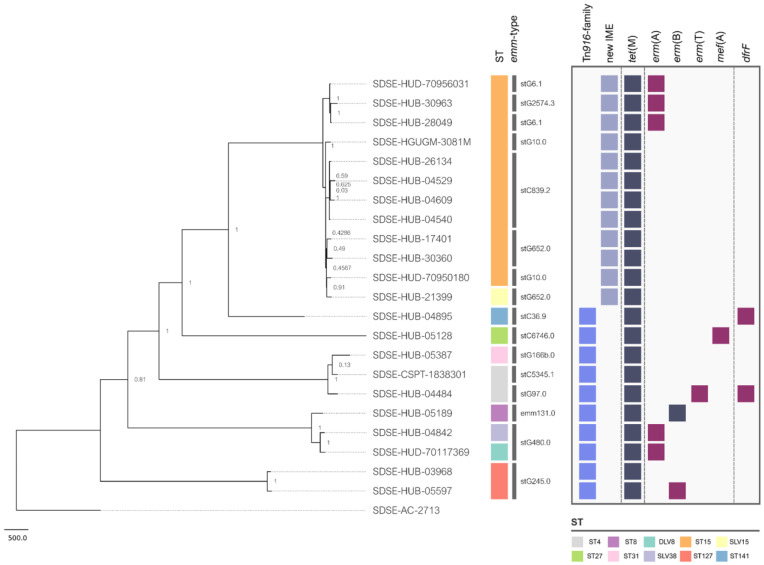
Phylogenetic tree and presence/absence matrix depicting the acquired resistance genes of iSDSE carrying *tet*(M). The strain names are shown on the tree branches. The colored squares in the first column represent the ST (Sequence-Type). The second column shows the *emm*-types. The two first columns of light blue and grey squares represent the mobile genetic elements (MGEs) in which the *tet*(M) gene is found. The following columns represent a presence/absence matrix of *tet*(M) and other acquired resistance genes indicating in dark blue when present in the same element as *tet*(M) and in dark red when present in a different element.

**Figure 2:**
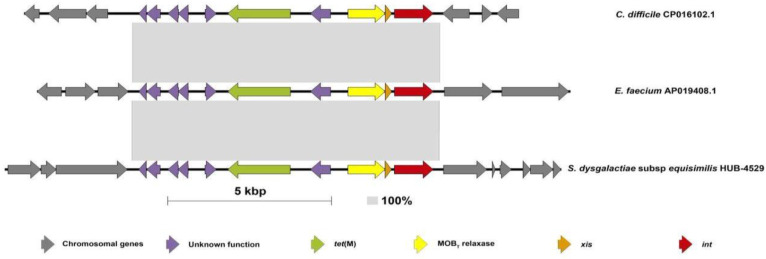
Schematic representation of the IME containing *tet*(M) in the SDSE compared with the sequence from *E. faecium* and *C. difficile*. This figure shows the structure of the IME containing the *tet*(M) gene found in twelve strains of the present study (HUB, HUD, HGUGM) compared with the previously described isolates in the literature for *E. faecium* (Acc. num AP019408.1) and *C. difficile* (Acc. num CP016102.1). The size of this IME is 9352 bp. The grey-shaded areas connect identical regions (100% identity). Each arrow represents a gene. Colors represent different genes. Grey: chromosomal genes (compared in SDSE with reference strain (AC-2713) (Acc num HE858529.1)). Red: integrase; orange: excisionase; yellow: MOBT relaxase; green: *tet*(M) gene; purple: unknown function genes.

The genome analysis showed that tetracycline resistance was associated with the presence of the *tet*(M) (*n* = 22), *tet*(W) (*n* = 1), or *tet*(L) plus *tet*(W) (*n* = 1) genes. This gene distribution among the iSDSE agreed with previous results from other regions [15,16]. Nevertheless, exploring the genetic environment of the *tet*(M) gene, we found that it was carried by two different types of elements transferable by conjugation. In ten isolates, the *tet*(M) gene was carried by an ICE belonging to the Tn*916*-family [Tn*916* (*n* = 9) and Tn*3872* (*n* = 1, also having the *erm*(B) gene)], as previously described. On the other hand, 12 isolates had the *tet*(M) gene in a new IME (IME_SDSE_HUB-4529; Figure 1 and Figure 2) that was identical (100% nucleotide identity) in all 12 isolates. The IME_SDSE_HUB-4529 encodes a tyrosine integrase and a relaxase of the MOBT superfamily. All of them are integrated in the same site located in an intergenic region between the genes encoding for the alpha subunit of ribonucleotide reductase of class Ib (aerobic) and the Na^+^-dependent branched-chain amino acid transporter (Figure 2). A BLASTN search identified identical IMEs (100% identity) in the *Enterococcus faecium* (Acc. num AP019408.1) and *Clostridioides difficile* (Acc. num CP016102.1) genomes. The insertion sites of the element in the three species were different suggesting that the integrase is not site-specific. This fact together with the 100% identity of the IMEs and with the clustering of the strains on the phylogenetic tree suggests the clonality of the 12 strains and a recent acquisition of the IME by a common ancestor to these strains. A further BLASTN search identified similar structures in other Gram-positive bacteria (*E. faecalis*, *C. innocuum,* and *Catenibacterium* sp.) commonly found in the gut microbiota with an identity above 95% (Supplementary Figure S1). The presence of IME structures in iSDSE with a putative origin from other bacterial species is a cause for concern, especially for *E. faecium* which is known to be associated with vancomycin and multidrug resistance. In fact, transfer of the *van*(A) gene from enterococcal species to *S. aureus* has already been described [17]. The chance of DNA exchanges is more plausible in the current scenario, with an increasing frequency of SDSE which is a cause of human infection.

Aside from the elements carrying tetracycline resistance, most of the isolates also had other resistance mechanisms carried by other elements (Figure 1). Among isolates carrying Tn*916,* one strain had the *erm*(T) gene in a non-self-transmissible plasmid and the *dfr*(F) in a different IME; two isolates had the Tn*916* inserted into a larger ICE belonging to the Tn*1549*-family that also had *erm*(TR); a single isolate had the *dfr*(F) in a different IME; one isolate had the macrolide efflux genetic assembly (MEGA) element carrying the *mef*(E) gene; and finally, one isolate also had *erm*(B) located near an insertion sequence. On the other hand, three out of 12 isolates carrying the IME_SDSE_HUB-4529 also had the *erm*(TR) gene located in a Tn*1549*-family of ICE.

Table 1 shows the antibiotic susceptibility of the tetracycline-resistant SDSE. Beta-lactams (penicillin and cefotaxime), vancomycin, and linezolid were active against all the strains. In fact, beta-lactams are the first-line treatment of SDSE infections, and linezolid could be used as an alternative treatment in case of allergy. Regarding levofloxacin, all strains were “susceptible, increased exposure” following the EUCAST criteria. Nevertheless, one strain had a D83A substitution in ParC and had a MIC of levofloxacin of 2 mg/L which, as has occurred in other streptococci, could be the first step to achieving high-level fluroquinolone resistance under antibiotic pressure. Other resistance rates could be explained by the transferable resistance mechanisms described above.

The phylogenetic analysis of the tetracycline-resistant isolates showed a high STs’ and *emm*-type diversity. We found 10 different STs (ST15 *n* = 11, ST4 *n* = 2, ST127 *n* = 2, and ST8, ST27, ST31, ST141, DLV8, SLV15, SLV38 *n* = 1), and 13 different *emm*-types (stC839.2 *n* = 4, stG652.0 *n* = 3, stG480.0 *n* = 2, stG6.1 *n* = 2, stG10.0 *n* = 2, stG245.0 *n* = 2, and stG2574.3, stG166b.0, stG6746.0, stC36.9, emm131.0, stC5345.1, stG97.0 *n* = 1) among tetracycline-resistant strains harboring the *tet*(M). These STs and *emm*-types have been previously described as a cause of disease in humans [15,16,18,19,20,21,22]. Figure 1 shows the phylogenetic relatedness of the SDSE strains carrying *tet*(M) regardless of the MGEs carrying this gene. Of note, the isolates carrying the *tet*(M) gene in the ICEs of the Tn*916* family were genetically more distinct (Figure 1) than the strains carrying *tet*(M) in the novel IME. In fact, all isolates carrying the IME_SDSE_HUB-4529 belonged to the same clonal complex (CC15) suggesting a clonal spread of resistance. Nevertheless, these strains of CC15 had different *emm*-types. In fact, no clear relationship between *emm*-type and clonal complex has been established for SDSE, as occurs for *S. pyogenes*, highlighting the more accurate role of CC to differentiate SDSE lineages. Some tetracycline-resistant isolates were also resistant to macrolides, lincosamides, and trimethoprim–sulfamethoxazole. These co-resistances were more frequent in the strains carrying Tn*916* (7/10) than in the ones that carry the IME_SDSE_HUB-4529 (3/12) (Figure 1).

MGEs are a frequent cause of resistance dissemination in streptococci. Among them, the ICEs had all the genes required for conjugation and could easily spread intra- and interspecies [23]. In this way, we found a high clonal diversity of tetracycline-resistant strains harboring the *tet*(M) gene in the Tn*916*. Furthermore, this element has been associated with resistance in streptococci since the late 1990s, enhancing the opportunities to spread among different strains and species [24]. In contrast, we found a high genetic relatedness among isolates having the *tet*(M) gene in the new IME (ST15), highlighting that the increase in the prevalence of this new element could be associated preferably to clonal spread of the successful lineages having them. Nevertheless, the presence of ICEs harboring other resistance genes in some of the strains suggests a putative scenario of co-dissemination among other streptococci. Furthermore, it is important to note that other ICEs lacking resistance determinants could also be found among strains and could play a role in the spreading of IMEs [11].

In conclusion, the spread of tetracycline resistance in *Streptococcus dysgalactiae* subsp. *equisimilis* is mediated by both the horizontal spread of mobile genetic elements among different strains and by the clonal spread of tetracycline-resistant lineages harboring *tet*(M). We described a new integrative mobilizable element in *Streptococcus dysgalactiae* subsp. *equisimilis* with a putative origin from *E. faecium* or *C. difficile*, which highlights the importance of interspecies transfer of resistance determinants.

## 3. Materials and Methods

This was a retrospective study from 2012 to 2019 including all tetracycline-resistant iSDSE in the Hospital Universitari de Bellvitge (HUB), an adult tertiary teaching hospital located in the Southern Barcelona area, Spain. Furthermore, in order to understand the spread of MGEs and tetracycline-resistant lineages around the country, isolates causing iSDSE in three other hospitals during 2018 were also included. These additional hospitals were in the Center (H. Universitario Gregorio Marañón, Madrid, HGUGM), North (H. Universitario Donostia, Donostia, HUD), and East (Consorci Sanitari Parc Taulí, Sabadell, CSPT) of Spain.

The strains were identified by Matrix-Assisted Laser Desorption/Ionization—Time of Flight (MALDI-TOF, Bruker-Daltonics, Bremen, Germany) and tested by both disc-diffusion and broth microdilution methods for antimicrobial susceptibility following the EUCAST recommendations and criteria (www.eucast.org (accessed on 10 October 2022)). For microdilution, the commercially available plates were used (STRHAE, Sensititre, ThermoFisher Scientific, Waltham, MA, USA). The following antibiotics were tested: penicillin, ampicillin, cefotaxime, erythromycin, clindamycin, linezolid, tetracycline, chloramphenicol, ciprofloxacin, levofloxacin, vancomycin, and trimethoprim–sulfamethoxazole.

The molecular characterization of tetracycline-resistant strains was performed by whole genome sequencing. Genomic DNA was extracted using a QIAamp DNA Mini Kit (Qiagen) and quantified with the QuantiFluor dsDNA System (Promega Corporation, Madison, WI, USA). The libraries were prepared using Nextera XT and sequenced by paired-end sequencing (2 × 150 bp) on an Illumina MiSeq Platform (Illumina Inc., San Diego, CA, USA), following the manufacturer’s instructions. The read quality assessment and genome assembly was conducted using the INNUca v4.2 pipeline (github.com/B-UMMI/INNUca (accessed on 10 October 2021)). The multilocus sequence type (MLST) was in silico deduced using the MLST v2.4 software (github.com/tseemann/mlst (accessed on 10 October 2021)) and the PubMLST database (pubmlst.org/organisms/streptococcus-dysgalactiae (accessed on 0 October 12021)) and the *emm*-type by the CDC databases (www2.cdc.gov/vaccines/biotech/strepblast.asp (accessed on 10 October 2021)). The final assemblies were annotated with Prokka v1.13.7, whereas genes related to conjugative or mobilizable genetic elements were annotated with ICEscreen (Lao et al., unpublished results), a tool to detect ICEs and integrative mobilizable elements (IMEs) in Firmicute genomes. The resistance genes were found using the ResFinder database [25]. The genetic environment of the *tet*(M) gene was studied through comparison with previously described sequences present in public databases using Geneious 9.1.7 (Biomatters, Auckland, New Zealand). For phylogenetic analysis, a single nucleotide polymorphism alignment with Snippy v4.4.0 (github.com/tseemann/snippy (accessed on 11 January 2023)) was performed, the recombination was removed by Gubbins v2.4 [26], and subsequently, a final phylogenetic tree was constructed with RAxML-NG and 100 bootstrap replicates [27], represented using Microreact (microreact.org/ (accessed on 11 January 2023)). The sequence comparisons between the new IME described in this work and those available on NCBI were displayed using Easyfig program. The sequence data were deposited in the European Nucleotide Archive under the project accession number PRJEB55810 (Supplementary Material Table S1).

## Figures and Tables

**Table 1:** The antibiotic susceptibility of 24 tetracycline-resistant SDSE strains.

Antibiotic	% Susceptible	MIC (50) [mg/L]	MIC (90) [mg/L]
Penicillin	100	<0.03	<0.03
Cefotaxime	100	<0.06	<0.03
Erythromycin	54.2	<0.25	>4
Clindamycin	58.3	<0.25	>0.5
Linezolid	100	<1	-
Tetracycline	0	-	>4
Chloramphenicol	100	2	2
Levofloxacin	100 *	<1	<1
Vancomycin	100	<0.5	<0.5
Trimethoprim-Sulfamethoxazole	91.7	<0.5/9.5	<0.5/9.5

* “Susceptible, increased exposure”.

## Data Availability

Sequence data were deposited in the European Nucleotide Archive under the project accession number PRJEB55810.

## Supplementary Materials

The following supporting information can be downloaded at: https://www.mdpi.com/article/10.3390/antibiotics12030579/s1, Table S1: Antibiotic resistance and mobile genetic elements in the 24 tetracycline-resistant strains. Figure S1; Schematic representation of the IME containing *tet*(M) in SDSE (SDSE-HUB-4529) compared with different sequences from *E. faecium*, *C. difficile*, *Clostridium innocuum*, *E. faecalis,* and *Catenibacterium* sp.
