# Supplementary material for: A New Integrative and Mobilizable Element Is a Major Contributor to Tetracycline Resistance in Streptococcus dysgalactiae subsp. equisimilis
Source: Antibiotics (Basel). 2023 Mar 15;12(3):579. doi: 10.3390/antibiotics12030579 (PMC10044688; doi:10.3390/antibiotics12030579)
Supplement: Supplementary file 1 [file antibiotics-12-00579-s001.zip › Supplementary Figure S1.pdf]

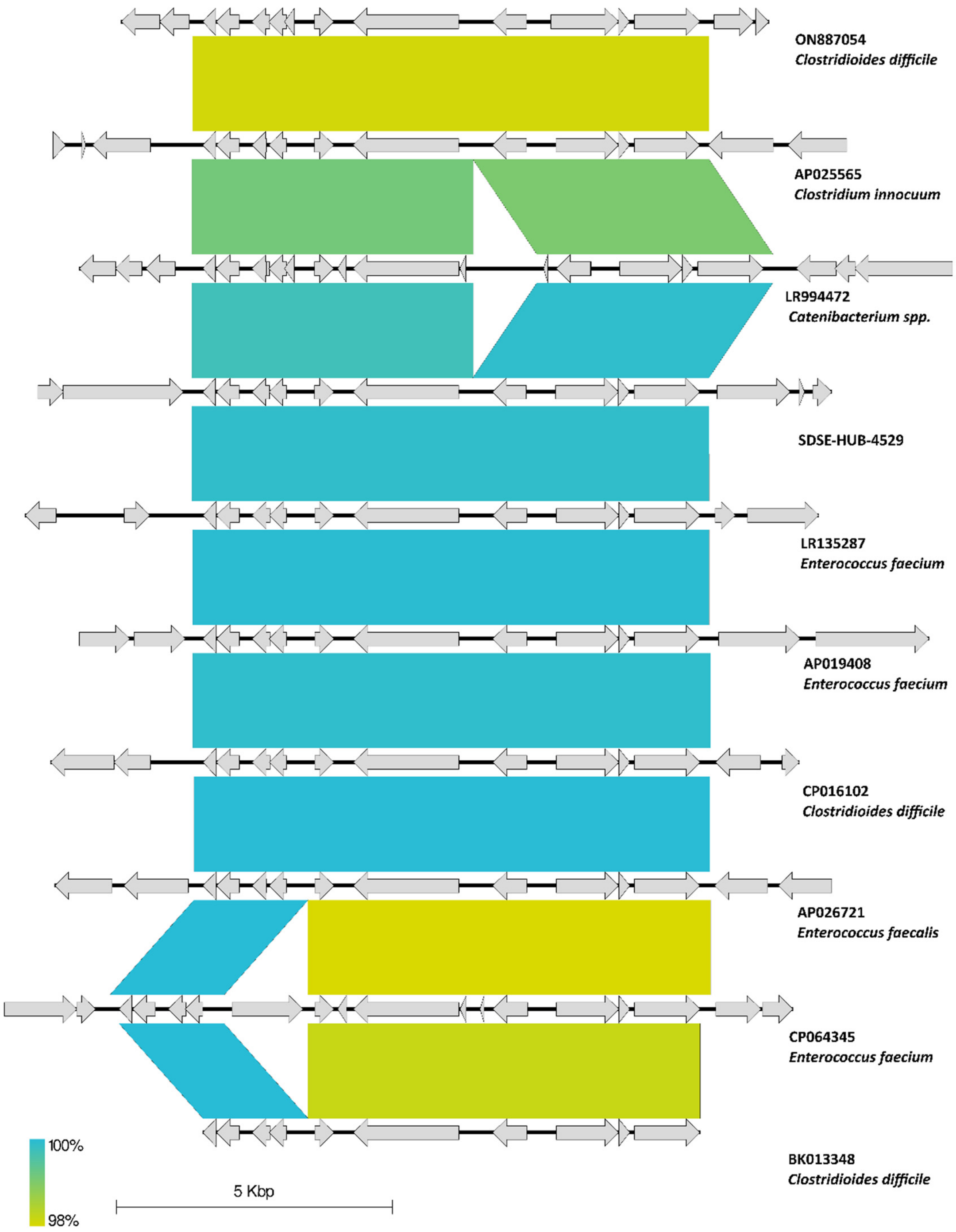

Figure S1. Schematic representation of the IME containing *tet*(M) in SDSE (SDSE-HUB-4529) compared with different sequences from *E. faecium*, *C. difficile*, *Clostridium innocuum*, *E. faecalis* and *Catenibacterium* sp. This figure shows that the IME structure of SDSE have 100% of identity with the same element of *E. faecium* and *C. difficile*, while the other species have an identity between 98-100% and insertions in the sequence. The shaded areas connect regions with different identity. Each arrow represents a gene. Accession numbers for each sequence are highlighted in boldface.
